# Jieduquyuzishen Prescription Attenuates Renal Fibrosis in MRL/lpr Mice via Inhibiting EMT and TGF-*β*1/Smad2/3 Pathway

**DOI:** 10.1155/2022/4987323

**Published:** 2022-05-10

**Authors:** Shan Wu, Lina Ji, Xuemin Fan, Sijia Fang, Jie Bao, Xiao Yuan, Yongsheng Fan, Guanqun Xie

**Affiliations:** ^1^Affiliated Hospital of Hangzhou Normal University, Hangzhou 310015, Zhejiang, China; ^2^The First Affiliated Hospital of Zhejiang Chinese Medical University, Hangzhou 310006, Zhejiang, China; ^3^Department of Medical Administration, Hangzhou Emergency Medical Center in Zhejiang Province, Hangzhou 310003, Zhejiang, China; ^4^Hangzhou TCM Hospital of Zhejiang Chinese Medical University, Hangzhou 310007, Zhejiang, China; ^5^School of Basic Medical Science, Zhejiang Chinese Medical University, Hangzhou 310053, Zhejiang, China; ^6^The Department of Endocrinology, The First Affiliated Hospital of Zhejiang Chinese Medical University, Hangzhou 310006, Zhejiang, China

## Abstract

Jieduquyuziyin prescription (JP) has been used to treat lupus nephritis (LN) and its effectiveness in the treatment of LN has been clinically proven, but the underlying mechanisms have yet to be completely understood. This aim of this study was to clarify the efficacy of JP on the epithelial-mesenchymal transition (EMT) of renal tubular epithelial cells and the molecular mechanisms of JP in MRL/lpr mice. In vivo, we observed the therapeutic actions of JP in MRL/lpr mice as well as its antifibrosis effect and potential mechanism. In vitro, we evaluated the role of JP in EMT and its possible mechanism through the EMT of human renal proximal tubular epithelial cells (HK-2) induced by transforming growth factor-beta 1 (TGF-*β*1) and M2c macrophages. HK-2 cells were treated with JP-treated serum, and MRL/lpr mice were treated by JP for 8 weeks. The results showed that JP alleviated disease activity, improved renal function, decreased proteinuria, and improved renal injury and fibrosis in MRL/lpr mice. Furthermore, JP suppressed the activation of the TGF-*β*1/Smad2/3 signaling pathway, upregulated the E-cadherin levels, and downregulated the Vimentin and mesenchymal *α*-smooth muscle actin (*α*-SMA) levels in the kidney of MRL/lpr mice. JP was further found to prevent the TGF-*β*1 and M2c macrophages-induced EMT of HK-2 cells. Collectively, JP could alleviate the disease activity of MRL/lpr mice, improve renal function, and attenuate renal fibrosis, and its underlying mechanisms may be related to the inhibition of EMT and TGF-*β*1/Smad2/3 signaling pathway.

## 1. Introduction

Systemic lupus erythematosus (SLE) is a refractory autoimmune disease that often involves multiple systems throughout the body, among which the kidney is one of the most common organs involved. Due to differences in gender, age, and ethnicity, approximately 40–70% of SLE patients have Lupus nephritis (LN) [1], which is the main risk factor for end-stage renal disease (ESRD) and death. Although the prognosis of LN patients has gradually improved in the past half-century [2], recent studies have shown that 10.1% of LN patients will progress to ESRD within 10 years of onset [3].

Antimalarials, immunosuppressants, and biological agents have been used to treat LN in recent years, and glucocorticoids (GC) is still the major current therapy for LN in the initial remission phase and the subsequent maintenance treatment phase [4]. Nevertheless, long-term use of GC may bring debilitating consequences to patients, including diabetes, cardiovascular disease, and osteoporosis and cause irreversible organ damage [5]. Therefore, there is an urgent need to develop new drugs with similar potent immune effects but no adverse metabolic effects.

Tubulointerstitial lesions, including inflammatory cell infiltration, tubular atrophy, and interstitial fibrosis, are independent risk factors for adverse renal outcomes of LN [6, 7]. Tubular epithelial cells (TECs) atrophy promotes immune cell infiltration and interstitial renal fibrosis; injured renal tubular epithelial cells have dysfunction, which makes them transition to a profibrotic phenotype [8]. The damage of TECs can lead to the loss of functional substance and escape survival mechanisms, such as the initiation of the epithelial-mesenchymal transition (EMT) program induced by transforming growth factor-beta 1 (TGF-*β*1) [9]. Studies have found that the phenotype of renal tubular epithelial cells changes during EMT. The expression level of epithelial markers such as E-Cadherin decreased, while the level of mesenchymal markers such as *α*-smooth muscle actin (*α*-SMA) and Vimentin increased [10]. The EMT of TECs is a sign of renal fibrosis and is a component of early functional kidney damage. By targeting to block EMT, the process of renal interstitial fibrosis can be inhibited [11]. Therefore, regulating EMT is the best choice to prevent progressive tubulointerstitial fibrosis [12].

Many factors may trigger EMT in TECs, such as TGF-*β*1, connective tissue growth factor, hypoxia, and basement membrane integrity [13–16]. In addition, macrophages play an important role in the process of inflammation and fibrosis of the kidney and promote the development of fibrosis [17]. It has been confirmed that infiltrating macrophages is related to the degree and severity of renal fibrosis [18]. Cell coculture experiments found that M2c macrophages can promote the EMT process of renal tubular epithelial cells [19]. TGF-*β*1 is a multifunctional cytokine that plays a fundamental role in regulating renal fibrosis. Meanwhile, TGF-*β*1 and its two receptors TGF-*β* receptor I and TGF-*β* receptor II play an important role in EMT and fibrosis. The main downstream mediators of TGF-*β*1 are Smad2 and Smad3, which are involved in EMT induced by TGF-*β*1 [20].

Traditional Chinese medicine (TCM) has the characteristics of multitarget and multichannel intervention, which may be a safe and effective treatment for the complex pathogenesis of SLE. Jieduquyuzishen prescription (JP) is composed of ten herbs including *Artemisia annua* L., *Cimicifuga heracleifolia Kom.*, *Hedyotis diffusa Willd*, *Paeonia suffruticosa Andr.*, *Trionyx sinensis Wiegmann*, *Centella asiatica* (L.) *Urb.*, *Citrus medica* L. *var. sarcodactylis Swingle*, *Glycyrrhiza uralensis Fisch*, *Paeonia lactiflora Pall.*, and *Rehmannia glutinosa Libosch.* It has been widely used in the treatment of lupus nephritis in China and has achieved good clinical effects [21]. Previous clinical studies have found that JP treatment can not only improve the condition with lupus nephritis, but also reduce the incidence of adverse reactions and complications during GC treatment [22]. Moreover, published animal studies have found that JP can effectively alleviate kidney damage in mice with LN [23].

To date, no research has focused on the effect of JP on renal fibrosis. This study was conducted *in vivo* (MRL/lpr mice) and *in vitro* (human renal proximal tubular epithelial cells (HK-2)) to evaluate the antikidney fibrosis effect of JP and its potential mechanism of action.

## 2. Materials and Methods

### 2.1. Preparation of JP

JP consists of ten crude drugs: dried aerial parts of *Artemisia annua* L.(12 g), rhizome of *Cimicifuga heracleifolia Kom*. (9 g), dried aerial parts of *Hedyotis diffusa Willd* (15 g), prepared root of *Paeonia suffruticosa Andr.* (12 g), carapace of *Trionyx sinensis Wiegmann* (12 g), dried aerial parts of *Centella asiatica (L.) Urb.* (15 g), fruit of *Citrus medica* L. *var. sarcodactylis Swingle* (9 g), rhizome of *Glycyrrhiza uralensis Fisch* (6 g), prepared root of *Paeonia lactiflora Pall.* (12 g), and prepared root of *Rehmannia glutinosa Libosch.* (15 g) (Table 1), corresponding to the common dose for adult humans, which was obtained from Zhejiang Chinese Medical University Medical Pieces., LTD (Hangzhou, China). After soaking in water (w/v, 1/10) for 1 h, the mixed herbs were boiled for 2 h for extraction. The residue was extracted again for another 2 h. The filtrates were collected, combined, and concentrated to 1.56 g crude drug/mL and then preserved at 4°C and rewarmed before administration.

### 2.2. Preparation of JP-Treated Rat Serum

Clean Sprague-Dawley male rats were provided by the Animal Experiment Center of Zhejiang University of Traditional Chinese Medicine, with an initial weight of 180–220g. The above SD rats were randomly divided into 3 groups, namely the control group (*n* = 6), the JP-treated group (*n* = 6), and the prednisone-treated group (*n* = 6). On the basis of dose conversion from human to rat according to clinical application, the dosage of JP was 1 ml/100g [24]. The treatment time was 5 days, and the rat blood was obtained after the last administration. After the collected blood was allowed to stand at room temperature for 2 hours, the blood was centrifuged at 3000 r/min for 15 minutes, and the serum was separated. The serum was then inactivated at 56°C for 30 minutes and filtered and stored in a refrigerator at −80 [25].

### 2.3. In Vitro

#### 2.3.1. Cell Culture and Drug Treatment

HK-2 cells were cultured in RPMI-1640 medium (100 U/ml penicillin and 100 *μ*l/ml streptomycin) supplemented with 10% fetal bovine serum (FBS) (Gibco, CA, USA) at 37°C in a humidified atmosphere with 5% CO2. Subsequently, the cells were cultured in a serum-free medium for 24 h and stimulated with TGF-*β*1 (10 ng/ml, PeproTech, USA) in the absence and presence of JP.

#### 2.3.2. Cell Viability Analysis

HK-2 cells in the logarithmic growth phase were seeded in 96-wells plates and cultured in a 37°C, 5% CO2 incubator for 24 h. HK-2 cells viability was tested by Cell Counting Kit-8 (CCK-8) (Beyotime, Shanghai, China). The cells were treated with JP-treated serum at concentrations of 0, 1, 2.5, 5, 7.5, 10, 15, 20, and 30% (v/v). Meanwhile, a blank control group is set; that is, no cells are inoculated, and the operation is the same as other wells. And then, the CCK8 reagent was added to the cells according to the instructions, and finally the absorbance was measured by a microplate reader (PerkinElmer, Enspire, MA, USA).

#### 2.3.3. Coculturing Experiments

Human monocytic leukaemia cell line THP-1 cells in logarithmic growth phase were collected to prepare cell suspension, and the cells concentration was adjusted to 5 × 10^5^ cells/mL. The cells were inoculated with 1 × 10^6^ cells per well in 6-well plates containing RPMI-1640 medium, 1% FBS, and 200 ng/ml phorbol 12-myristate 13-acetate (PMA, Sigma Aldrich, USA) for 12 h. These cells were then treated with 20 ng/ml IL-10 for 0 h, 12 h, and 24 h [19]. The mRNA expressions of iNOS, CD163, and CD206 were detected by RT-qPCR to verify the induction of M2c macrophages.

Similarly, THP-1 cells were induced to differentiate into M2c macrophages in the Transwell plate (Corning, NYC, USA). And HK-2 cells were pipetted into Transwell upper chamber (0.4 *μ*m pore size; Corning, USA). When the HK-2 cells adhered to the wall, the cells were rinsed once with PBS and the medium was renewed. Then, the Transwell upper chamber was put into the Transwell plate containing M2c macrophages that were 10 times more than the HK-2 cells in cell number and sent to an incubator (37°C, 5% CO_2_). Subsequently, the cells were treated with JP and prednisone-treated serum.

### 2.4. In Vivo

#### 2.4.1. Mice and Treatments

MRL/lpr and MRL/Mp mice (female, 6–8 weeks) were obtained from SLAC Laboratory Animal Co., Ltd. (Shanghai, China) and housed in a SPF laboratory under standard temperature (25°C) and humidity (40–60%) conditions with a 12 h light/dark cycle and standard pallet diet and water.

MRL/lpr mice were randomly distributed into three groups: mice treated with diluted water (Model group, *n* = 10), mice treated with JP (JP group, *n* = 10), and mice treated with prednisone (PDN group, *n* = 10). And MRL/MP mice were treated with diluted water (Control group, *n* = 10). From the age of 8–16 weeks, mice of the JP group were administered JP (18 ml/kg body weight (bw) per day, i.g.) [26] and mice in the PDN group were intragastrically administered 5 mg/kg of prednisone suspension per day, which is equivalent to 0.5 mg/kg in humans. The model group and the control group were treated with an equal volume of diluted water. Mouse urine was collected once a week for urine protein determination. Mouse serum was collected from the orbital venous plexus before sacrifice, and mouse kidney tissue samples were obtained immediately after the sacrifice. All animal experiments were conducted in accordance with the National Institutes of Health's guidelines for laboratory animal care and use. All the above animal studies were approved by the Animal Experiment Ethics Committee of Zhejiang Chinese Medical University.

#### 2.4.2. HE Staining and Masson Staining

For the examination of the renal histopathological changes, the kidney samples from the mice in the different experimental groups were fixed in 4% buffered paraformaldehyde for 48 h, dehydrated, and then embedded in paraffin. The tissue sections of 4 *μ*m thickness were sliced from each embedded sample and stained with hematoxylin and eosin (H&E) or Masson's trichrome according to standard protocol.

#### 2.4.3. Immunohistochemistry (IHC)

The paraffin-embedded kidney sections were deparaffinized in xylene and rehydrated through a descending gradient of ethanol. The sections were then incubated in primary antibody overnight at 4°C and then washed three times in PBS (PH 7.4) for 5 min on each occasion. The primary antibodies used in the analysis were as follows: anti-E-Cadherin (1 : 200 dilution) and anti-*α*-SMA (1 : 100 dilution). An appropriate secondary antibody (anti-rabbit) was incubated with the slides at 37°C for 20 min. Optical microscopy (Motic, Xiamen, China) was employed for image acquisition.

### 2.5. Enzyme-Linked Immunosorbent Assay (ELISA)

We measured the levels of protein and creatinine in urine. The urine of the mice was centrifuged at 12,000 r/m for 15 min to collect the supernatant. The concentration of protein and creatinine in the supernatant was determined using a suitable ELISA kit (Jiancheng, Nanjing, China).

Each mouse venous blood was allowed to stand for 30 min, and the serum was collected by centrifugation. Afterwards, the concentration of anti-dsDNA, anti-nRNP/Sm, and ANA in the serum were measured with the corresponding ELISA Kit (EUROIMMUN, Lubeck, Germany). The procedure was strictly in accordance with the kit instructions and the required indicators were measured in a microplate reader (PerkinElmer, Enspire, MA, United States).

### 2.6. Total RNA Extraction and Quantitative Real-Time PCR (RT-qPCR)

Extraction of total RNA from cells and kidney tissues was used by RNAiso Plus (TAKARA, Dalian, China) and then was transformed into complementary DNA using the TAKARA Reverse Transcription System Kit (TAKARA, Dalian, China). The Roche Light Cycler 96 SW1.1 instrument (Roche, Basel, Switzerland) was used for quantitative real-time polymerase chain reaction (Rt-qPCR). The results were analyzed using 2^(-△△CT)^ values. GAPDH (Sangon Biotech, Shanghai, China) was used as a control.  Table 2 shows the sequences of the required primers in the experiment.

### 2.7. Western Blot Analysis

Extractions of total protein from different groups of cells and kidney tissues were used by Qproteome Mammalian Protein Prep Kit (QIAGEN, Dusseldorf, Germany). The protein concentrations were detected by BCA kit (Biosharp, Hefei, China). The isolated equivalent amount of PVDF membranes were incubated overnight after adding E-Cadherin (Cell Signaling Technology, 3195, 1 : 1,000), *α*-SMA (Cell Signaling Technology, 19245, 1 : 1,000), Vimentin (Cell Signaling Technology, 5741, 1 : 1,000), TGF-*β*1 (Abcam, ab179695, 1 : 1,000), and Smad2/3 (Cell Signaling Technology, 8685, 1 : 1,000), individually. The membranes were then incubated with anti-rabbit IgG (1 : 2,000) for 1 h and chemically developed the membranes with ECL Substrate (Bio-Rad, CA, United States). The signals were quantified by an imager (ProteinSimple, CA, United States), and ImageJ software was used to quantify protein bands as a ratio to *α*-tubulin and GAPDH.

### 2.8. Statistical Analyses

All data were expressed as mean ± standard deviation. The statistical significance was determined by Student's *t*-test and one-way ANOVA analysis. Differences were considered to be significant when *P* < 0.05.

## 3. Results

### 3.1. Effects of JP-Treated Serum on the Viability of HK-2 Cells

**Figure 1:**
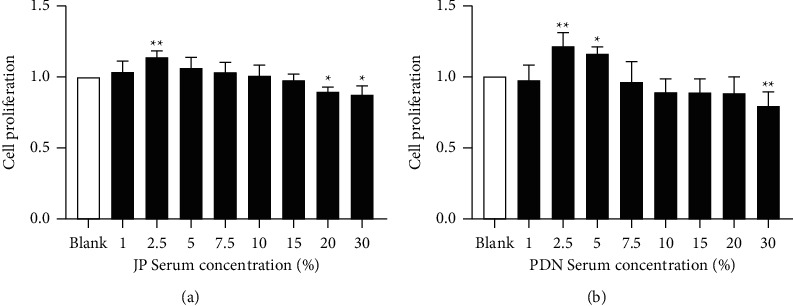
The effect of (a) JP-treated serum and (b) PDN-treated serum for 24 h on the viability of HK-2cells. Data were shown as the mean ± SD (*n* = 6) of one representative experiment. ^*∗*^*P* < 0.5 and ^*∗∗*^*P* < 0.01 versus Blank group.

CCK-8 method was used to investigate the effects of different concentrations of JP-treated serum on HK-2 cells. Compared with the control group, 2.5% (v/v) of JP- treated serum had the most significant effect on the viability of HK-2 cells (Figure 1(a)). PDN-treated serum had the same effect on cell viability as JP-treated serum (Figure 1(b)). Similarly, the control group selected the corresponding drug-containing serum with a concentration of 2.5% (v/v) for *in vitro* intervention.

### 3.2. JP-Treated Serum Inhibited the EMT Process of HK-2 Cells Stimulated by TGF-*β*1

**Figure 2:**
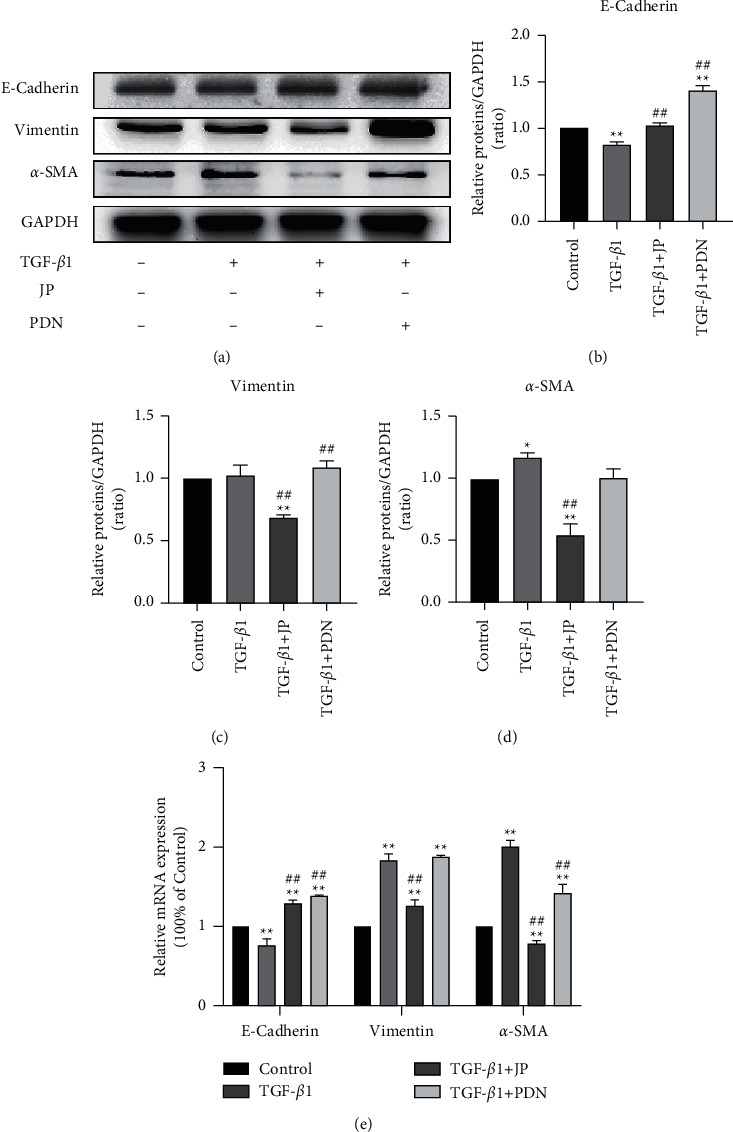
JP-Treated Serum inhibited the EMT process of HK-2 cells stimulated by TGF-*β*1. (a) The protein bands of E-Cadherin, Vimentin and *α*-SMA in HK-2 cells stimulated by TGF-*β*1. (b–d) Quantitative analysis of protein expression alteration HK-2 cells stimulated by TGF-*β*1. (e) The mRNA expressions of E-Cadherin, Vimentin and *α*-SMA in HK-2 cells stimulated by TGF-*β*1. Data were expressed as mean ± SD (*n* = 3). ^*∗*^*P* < 0.05 and ^∗∗^*P* < 0.01, compared with the Control group; ^##^*P* < 0.01, compared with the TGF-*β*1 group.

In order to determine whether JP-treated serum can affect the EMT process of HK-2 cells stimulated by TGF-*β*1 *in vitro*, we used JP-treated serum to intervene the TGF-*β*1-stimulated HK2 cells and detected the expression of E-Cadherin, Vimentin, and *α*-SMA 24 h later. Western blot results showed that the expression levels of Vimentin and *α*-SMA were significantly increased in HK-2 cells stimulated by TGF-*β*1 for 24 h (*P* < 0.01) (Figures 2(c) and 2(d)), while the expression level of E-Cadherin was significantly decreased (*P* < 0.01) (Figure 2(b)). These suggested that TGF-*β*1 stimulation can promote the EMT process of HK-2 cells. Meanwhile, the administration of JP-treated serum downregulated the high expression of Vimentin and *α*-SMA in HK-2 cells stimulated by TGF-*β*1 and increased the expression of E-cadherin in HK-2 cells (*P* < 0.01) (Figures 2(b)–2(d)). RT-qPCR confirmed that 24 h after TGF-*β*1 stimulation, the transcription levels of Vimentin and *α*-SMA in HK-2 cells were significantly increased, and the transcription levels of E-Cadherin were significantly reduced (*P* < 0.01) (Figure 2(e)). JP-treated serum inhibited the upregulation of the transcription levels of Vimentin and *α*-SMA in HK-2 cells stimulated by TGF-*β*1 and increased the transcription level of E-Cadherin (*P* < 0.01) (Figure 2(e)).

### 3.3. JP-Treated Serum Inhibited the EMT Process of HK-2 Cells Induced by Coculture of M2c Macrophages

**Figure 3:**
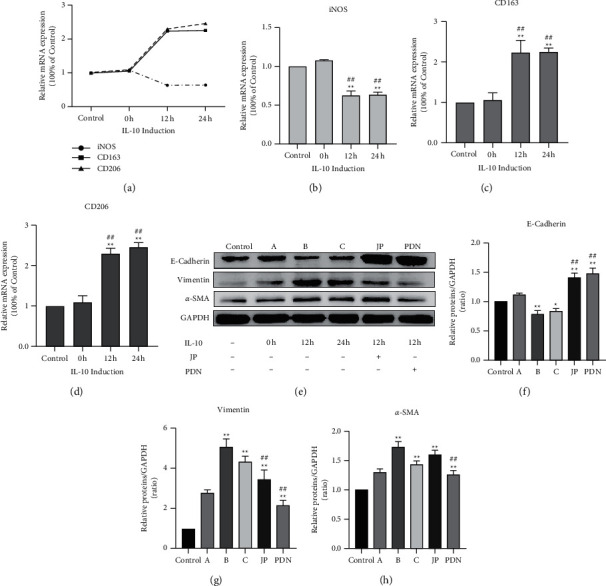
JP-treated serum inhibited the EMT process of HK-2 cells induced by coculture of M2c macrophages. (a) The mRNA expression trend of iNOS, CD163 and CD206 in THP-1 cells after IL-10 induction. (b–d) Bar graphs depict the fold changes of mRNA expression of iNOS, CD163 and CD206 assessed by quantitative RT-qPCR. (a–d) In the graphs, the THP-1 cells untreated with IL-10 as control group, and 0 h, 12 h and 24 h group represent the THP-1 cells induced with IL-10 for 0 h 12 h and 24 h respectively (^*∗∗*^*P* < 0.01 compared with the Control group; ^##^*P* < 0.01 compared with the 0 h group; n = 3). (e) The protein bands of E-Cadherin, Vimentin and *α*-SMA in HK-2 cells induced by coculture of M2c macrophages. ((f)-(h)) Quantitative analysis of protein expression alteration HK-2 cells induced by coculture of M2c macrophages. ^*∗∗*^*P* < 0.01, compared with the Control group; ^##^*P* < 0.01, compared with the B group; *n* = 3.

The THP-1 cells induced with IL-10. RT-qPCR results showed that after 12 hours of inducing, CD163 and CD206 mRNA expression increased significantly compared to the control group (*P* < 0.01) (Figures 3(a), 3(c), and 3(d)). In contrast, iNOS mRNA expression decreased after 12 hours of induction (*P* < 0.01) (Figures 3(a) and 3(b)). The features of THP-1 cells after 12 hours of inducing were consistent with the typical characteristics of polarized M2c macrophages. Subsequently, the HK-2 cells coculture of macrophages was treated with JP and prednisone-treated serum.

In order to determine whether JP-treated serum can affect the EMT process of HK-2 cells induced by M2c macrophages *in vitro*, we used drug-treated serum in the HK2 cells cocultured with M2c macrophages induced with IL-10 for 12 h and detected the expression of E-Cadherin, Vimentin, and *α*-SMA. Western blot results showed that after IL-10 stimulation for 12 or 24 h, the expression levels of Vimentin and *α*-SMA in the cocultured HK-2 cells increased significantly (*P* < 0.01) (Figures 3(e), 3(g) and 3(h)), while the expression level of E-Cadherin was significantly decreased for 12 h (*P* < 0.01) (Figures 3(e) and 3(f)). These indicated that M2c macrophages can promote the EMT process of HK-2 cells. Meanwhile, the administration of JP-treated serum could decrease the high expression of Vimentin and *α*-SMA in HK-2 cells after coculture and increase the expression of E-Cadherin (Figures 3(f)–3(h)).

### 3.4. JP-Treated Serum Inhibited TGF-*β*1/Smad2/3 Signaling Pathway in HK-2 Cells

**Figure 4:**
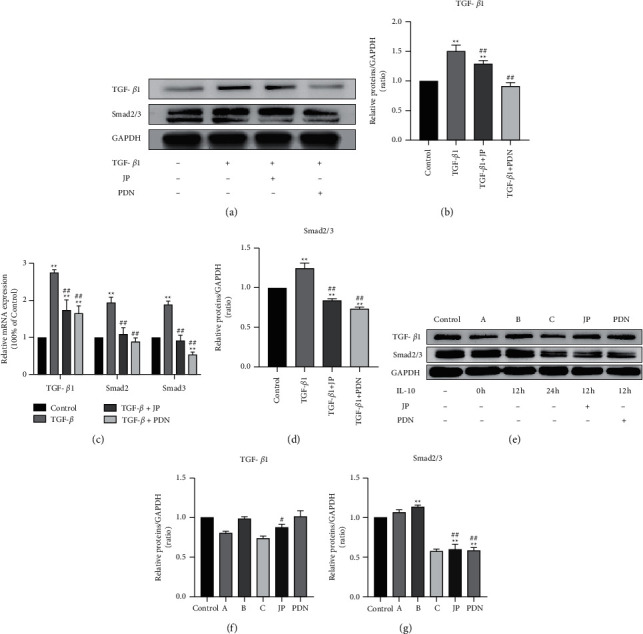
JP-Treated Serum inhibited TGF-*β*1/Smad2/3 signaling pathway in HK-2 cells *in vitro*. (a) The protein bands of TGF-*β*1 and Smad2/3 in HK-2 cells stimulated by TGF-*β*1. (b, d) Quantitative analysis of protein expression alteration HK-2 cells stimulated by TGF-*β*1. ^*∗∗*^*P* < 0.01, compared with the Control group. ^##^*P* < 0.01, compared with the TGF-*β*1 group; n = 3. (c) The mRNA expressions of TGF-*β*1, Smad2 and Smad3 in HK-2 cells stimulated by TGF-*β*1. (e) The protein bands of TGF-*β*1 and Smad2/3 in HK-2 cells induced by coculture of M2c macrophages. (f, g) Quantitative analysis of protein expression alteration HK-2 cells induced by coculture of M2c macrophages. ^*∗∗*^*P* < 0.01, compared with the Control group; ^#^*P* < 0.05 and ^##^^*∗∗*^*P* < 0.01, compared with the B group; *n* = 3.

We measured the levels of TGF-*β*1 and Smad2/3 in HK-2 cells after TGF-*β*1 stimulation and M2c-type macrophage coculture stimulation. The former western blot and RT-qPCR results showed that TGF-*β*1 levels were significantly increased in the HK-2 cells stimulated by TGF-*β*1 compared with the control group (*P* < 0.01) (Figures 4(a)–4(d)). The latter western blot results showed that Smad2/3 levels were significantly increased in the HK-2 cells induced by coculture of M2c macrophages compared with the control group (*P* < 0.01) (Figures 4(e)–4(g)). However, both levels of TGF-*β*1 and Smad2/3 in HK-2 cells treated with JP-treated serum were decreased (*P* < 0.01) (Figure 4).

### 3.5. JP Alleviated Renal Tissue Damage and Fibrosis in MRL/lpr Mice

**Figure 5:**
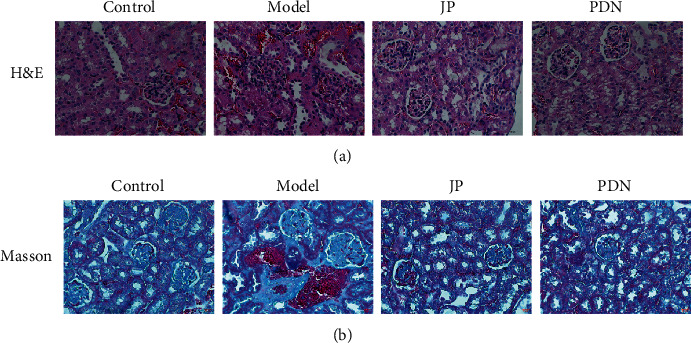
The treatment with JP attenuated renal lesions and fibrosis of MRL/lpr mice. (a, b) Kidney sections of the four groups were subjected to HE staining and Masson staining. Representative micrographs showed that JP ameliorated kidney injury characterized by glomerular swelling, renal interstitial fibrosis, tubular epithelial cells vacuolization, tubular dilatation, tubular atrophy, and inflammatory cell infiltration. Scale bar, 50 *μ*m. Magnification 40×.

In order to observe the effect of JP on renal histopathology, we performed H&E and Masson to observe the renal changes of mice. H&E staining results showed glomerular swelling, mesangial cell proliferation, mesangial matrix proliferation, and renal interstitial vascular dilation and congestion, accompanied by a large number of inflammatory cell infiltration in the model group mice (Figure 5(a)). However, in the JP group, the proliferation of mesangial cells decreased, and inflammatory cell infiltration was alleviated (Figure 5(a)). Masson staining showed that the expression of renal interstitial collagen was reduced in the JP group compared with the model group, and the renal tubular damage was reduced, including the relatively regular arrangement of the renal tubules and the decreased renal tubular atrophy and expansion (Figure 5(b)).

### 3.6. JP Protected Renal Function and Inhibited Disease Activity in MRL/lpr Mice

**Figure 6:**
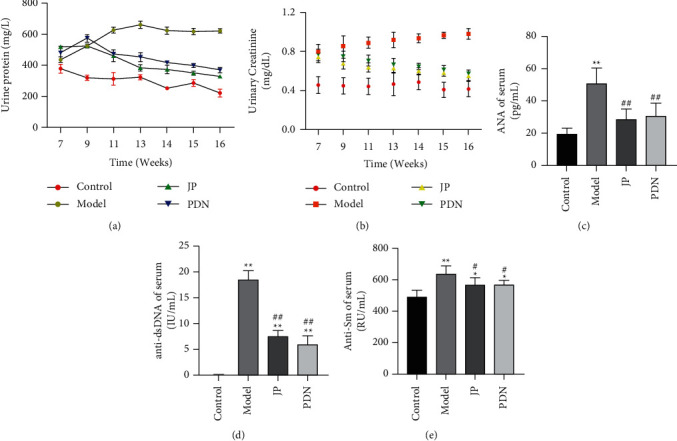
JP could protect renal function and inhibit disease activity in MRL/lpr mice. The dynamic changes of urinary protein (a) and urinary creatinine (b) concentrations in MRL/lpr mice during 7–16 weeks of test. The antibody titer of ANA (c), anti-dsDNA (d), and anti-Sm (e) in serum after 8 weeks of treatment. ^*∗*^*P* < 0.05 and ^*∗∗*^*P* < 0.01, compared with the Control group; ^#^*P* < 0.05 and ^##^*P* < 0.01, compared with the Model group; *n* = 10.

In order to clarify the efficacy of JP, we used ELISA to detect the concentrations of urinary creatinine and protein, as well as the contents of ANA, anti-dsDNA, and anti-nRNP/Sm in serum. The results showed that after the intervention of JP, the concentrations of urinary creatinine and protein in MRL/lpr mice decreased significantly (Figures 6(a) and 6(b)). Meanwhile, the contents of serum ANA (*P* < 0.01) (Figure 6(c)), anti-dsDNA (*P* < 0.01) (Figure 6(d)), and anti-Sm (*P* < 0.05) (Figure 6(e)) were also significantly decreased.

### 3.7. JP Inhibited the Process of Renal EMT in MRL/lpr Mice

**Figure 7:**
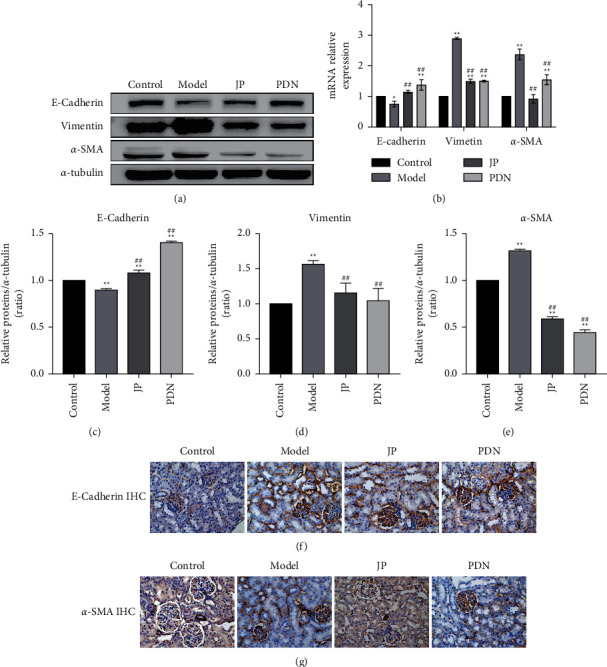
JP inhibited the process of renal EMT in MRL/lpr mice. (a) The protein bands of E-Cadherin, Vimentin and *α*-SMA in the renal. (b) The mRNA expressions of E-Cadherin, Vimentin and *α*-SMA in the renal. (c–e) Graphic presentation of western blot analyses in the four groups as indicated. (f) Representative micrographs showed that the down-expression of E-cadherin in model group was increased by JP. Scale bar, 20 *μ*m. (g) Representative micrographs showed that the up-expression of *α*-SMA in model group was decreased by JP. Scale bar, 20 *μ*m. ^*∗*^*P* < 0.05 and ^*∗∗*^*P* < 0.01, compared with the Control group; ^##^*P* < 0.01, compared with the Model group; n = 3. Magnification ((f), (g) are 40×).

In order to determine the effect of JP on the process of renal EMT in MRL/lpr mice, we evaluated the expressions of E-Cadherin, Vimentin, and *α*-SMA by western blot and RT-qPCR. Western blot results showed that the expression levels of E-Cadherin in JP group were significantly increased compared with the model group (*P* < 0.01) (Figures 7(a) and 7(c)), while the expression levels of Vimentin and *α*-SMA were significantly decreased (*P* < 0.01) (Figures 7(a), 7(d), and 7(e)). The results of RT-qPCR were consistent with the results of western blot (*P* < 0.01) (Figure 7(b)). Subsequently, IHC staining was performed on the kidney to observe the expression levels of *α*-SMA and E-Cadherin, which was consistent with the previous experimental results (Figures 7(f) and 7(g)).

### 3.8. JP Downregulated the TGF-*β*1/Smad2/3 Signaling Pathway in the Renal of MRL/lpr Mice

**Figure 8:**
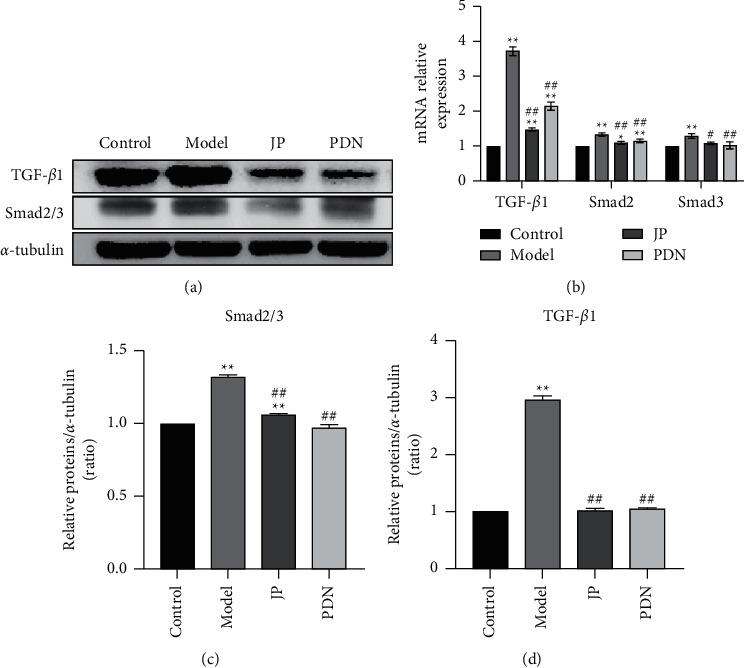
JP downregulated the TGF-*β*1/Smad2/3 signaling pathway in the renal of MRL/lpr mice. (a) The protein bands of TGF-*β*1 and Smad2/3 in the renal. (b) The mRNA expressions of TGF-*β*1, Smad2 and Smad3 in the renal. (c, d) Graphic presentation of western blot analyses in the four groups as indicated. ^∗∗^*P* < 0.01, compared with the Control group; ^#^*P* < 0.05 and ^##^*P* < 0.01, compared with the Model group; *n* = 3.

In order to evaluate the effect of JP on TGF-*β*1/Smad2/3 signaling pathway, we further studied the expression of TGF-*β*1 and Smad2/3 by western blot and RT-qPCR. The results showed that the protein expression levels of TGF-*β*1 and Smad2/3 in the model group were significantly higher than those in the control group (*P* < 0.01) (Figures 8(a), 8(c) and 8(d)), suggesting that the TGF-*β*1/Smad2/3 signaling pathway was activated in MRL/lpr mice. We found that JP could inhibit the protein expressions of TGF-*β*1 and Smad2/3 in MRL/lpr mice (Figures 8(a), 8(c) and 8(d)), thereby inhibiting the activation of TGF-*β*1/Smad2/3 signaling pathway. The results of RT-qPCR and western blot were consistent (*P* < 0.01) (Figure 8(b)).

## 4. Discussion

Although treatment strategies for LN have improved over the past twenty years, the rate of patients who progress to ESRD remains at about 10% [27]. Renal fibrosis is a common pathological manifestation of different types of chronic kidney disease. It is characterized by excessive accumulation and deposition of extracellular matrix (ECM), which leads to continuous scar tissue formation and progressive loss of renal function. Studies have shown that pathways involved in ECM protein synthesis in renal TECs in patients with LN are significantly upregulated, which is negatively correlated with treatment response [28]. Fibrosis in a short period of time is a repair mechanism after tissue injury, while continued fibrosis after severe or repeated damage will cause connective tissue to replace renal functional units and cause renal failure [29–31]. Complete remission or low disease activity is the treatment goal of LN [32], but there are still persistent inflammation and chronic damage in the kidney pathology of patients who reach the standard treatment [33]. Therefore, in addition to controlling or alleviating renal inflammation, it is urgent to prevent the process of chronic kidney disease. However, until now, there is no specific treatment to stop the progression of renal fibrosis.

In the course of clinical treatment, JP showed significant renal protection effect [21, 22]. Previous animal experiments have found that JP inhibits the TLR9 signaling pathway in the kidney [34], exerts its anti-inflammatory effect, and reduces kidney damage. In this study, we evaluated the effect of JP on disease improvement and renal protection in MRL/lpr mice. The results showed that JP alleviated disease activity; improved renal function, decreased proteinuria; and improved renal injury, fibrosis, and ECM deposition in MRL/lpr mice. Further studies have also shown that the underlying mechanism of its action is that JP inhibits the EMT and TGF-*β*1/Smad2/3 signaling pathways.

EMT is a process in which epithelial cells lose their epithelial expression and acquire a mesenchymal phenotype. The study found that the renal tubular epithelial cells, which could express the markers and characteristics of myofibroblasts *in vitro* after appropriate stimulation, did not participate in the transformation of mesenchymal myofibroblasts *in vivo* [35]. And the latter is directly involved in the synthesis of ECM protein, causing the accumulation of ECM and accelerating the process of fibrosis. Recent studies have put forward a new view of EMT; that is, renal tubular epithelial cells undergo phenotypic transformation after being injured and express markers of epithelial cells and mesenchymal cells at the same time, but do not leave the renal tubules [14]. By specifically blocking the EMT process in vivo, renal interstitial fibrosis can be effectively reduced [11]. Although EMT remains a controversial topic, there is consensus that it plays an important role in renal fibrosis [14, 36]. In this study, MRL/lpr mice successfully induced tubular EMT, and JP treatment alleviated this process by upregulating E-cadherin and downregulating *α*-SMA and Vimentin. Interestingly, *in vitro* cell experiment achieved consistent results. Therefore, the renal protective effect of JP may be related to the inhibition of EMT.

In the process of EMT, epithelial cells adapt to the stimulation of the immune microenvironment, thereby changing the plasticity phenotype. Macrophages in the immune microenvironment are considered to be the main part of driving kidney inflammation and fibrosis, because they synthesize and secrete a variety of molecules that promote or participate in the process of kidney fibrosis, such as growth factors, enzymes, and matrix proteins [37, 38]. Among them, TGF-*β* secreted by macrophages is considered to be one of the main factors that promote fibrosis [39]. M1 and M2 are the two main phenotypes of macrophages, and M1 macrophages are proinflammatory cell phenotypes, which can aggravate kidney damage while clearing the infection. The M2 type macrophages are anti-inflammatory cell phenotypes and play a repairing role [36]. M2 macrophages can be further divided into three subtypes according to their phenotype and function. Compared with M2a and M2b type macrophages, M2c type macrophages are more inclined to exert anti-inflammatory and repair effects in vivo. They are believed to reduce tissue damage and promote fibrosis [40, 41]. Previous studies have reported that the polarization characteristics of M2c type macrophages are high expression of CD163 and CD206, while iNOS, which has the polarization characteristics of M1 type macrophages, has low expression [42, 43]. According to previous studies [19], we used IL-10 to induce THP-1 cells for 12h and 24h and simultaneously detected the mRNA transcription levels of CD163, CD206, and iNOS. The results confirmed that we successfully induced M2c macrophages. Meanwhile, in our research results, M2c type macrophages successfully promoted the EMT process of renal tubular epithelial cells, which is consistent with the results of previous studies [19]. We also found that JP could also inhibit the EMT process of HK-2 cells induced by M2c macrophages.

TGF-*β*1/Smad2/3 signaling pathway is considered to be a key signaling pathway in promoting fibrosis. TGF-*β*1 binds to its receptor, activates Smad2 and Smad3, and then binds to Smad4 to form the Smad complex, which metastasizes to the nucleus and suppresses expression of epithelial marker proteins (e.g., E-Cadherin) and induces expression of mesenchymal marker proteins (e.g., *α*-SMA, Vimentin) to facilitate the EMT process [9, 44].

However, this study also had some shortcomings. Our previous research found that JP was consisted of a variety of active ingredients such as paeoniflorin and ferulic acid, both of which were discovered in JP freeze-dried powder and JP-treated serum [45]. However, the interaction between the active components in JP is not clear. Therefore, it is necessary to conduct further studies, and independent control tests should be carried out on the compound and each active ingredient. TGF-*β*1/Smad2/3 signaling knockout or specific blocking was not used as controls in this study. It cannot be proved that TGF-*β*1/Smad2/3 signaling pathway is the only mechanism of JP to inhibit EMT and improve renal fibrosis. Therefore, we will explore these questions in more depth to make the experiment more accurate and meaningful.

## 5. Conclusions

In conclusion, our study showed that JP alleviated renal inflammation, reduced proteinuria, improved renal function, and reduced renal fibrosis in MRL/lpr mice. In addition, its potential mechanism might be related to the inhibition of EMT and TGF-*β*1/Smad2/3 signaling pathway. Therefore, JP might be an alternative therapy that could both alleviate the disease of LN and improve renal fibrosis.

## Figures and Tables

**Table 1:** The compositions of Jieduquyuzishen prescription (JP).

Chinese name	Scientific name	Latin name	Weight (g)	Parts used
Qing Hao	Artemisiae annuae herba	*Artemisia annua* L.	12	Herb
Shen Ma	Cimicifugae rhizoma	*Cimicifuga heracleifolia Kom*.	9	Rhizome
Bai Hua She She Cao	Herba Hedyotis diffusae	*Hedyotis diffusa Willd*	15	Herb
Mu Dan Pi	Moutan cortex	*Paeonia suffruticosa Andr.*	12	Root
Zhi Bie Jia	Trionycis carapax	*Trionyx sinensis Wiegmann*	12	Tergum
Ji Xue Cao	Centellae herba	*Centella asiatica (L.) Urb.*	15	Herb
Fo Shou	Citri sarcodactylis fructus	*Citrus medica* L. *var. sarcodactylis Swingle*	9	Fruit
Sheng Gan Cao	Glycyrrhizae radix et rhizoma	*Glycyrrhiza uralensis Fisch*	6	Root
Ci Shao	Paeoniae radix rubra	*Paeonia lactiflora Pall.*	12	Root
Gan Di Huang	Rehmanniae radix	*Rehmannia glutinosa Libosch.*	15	Root Tuber

**Table 2:** Primers used for RT-qPCR.

Gene	Forward sequence	Reverse sequence
Human TGF-*β*1	CAGCAACAATTCCTGGCGATAC	TCAACCACTGCCGCACAACT
Mouse TGF-*β*1	CCGCAACAACGCCATCTATG	CTCTGCACGGGACAGCAAT
Human E-cadherin	AACAGGATGGCTGAAGGT	GGGCTTGTTGTCATTCTG
Mouse E-cadherin	GACAACGCTCCCATCCCA	CCACCTCCTTCTTCATCATAG
Human *α*-SMA	TCTCTCTATGCCTCTGGACG	ACAATCTCACGCTCAGCAGT
Mouse *α*-SMA	CTGAAGAGCATCCGACACTG	AGAGGCATAGAGGGACAGCA
Human Vimentin	ACCGCTTTGCCAACTACAT	TTGTCCCGCTCCACCTC
Mouse Vimentin	GGAGTCAAACGAGTACCGGA	GTGACGAGCCATCTCTTCCT
Human Smad2	GTCTGCC TTCGGTATTCTGC	GCTGCTCTTCTG GCTCAGTC
Human Smad3	AACAACCAGGA GTTCGCTGC	GGACCTTGTCAAGCC ACTGC
Mouse Smad2	ATGTCGTCCATCTTGCCATTC	AACCGTCCTGTTTTCTTTAGCTT
Mouse Smad3	CCCCCACTGGATGACTACAG	TCCATCTTCACTCAGGTAGCC
iNOS	GCAGAATGTGACCATCATGG	ACAACCTTGGTGTTGAAGGC
CD163	CGGCTGCCTCCACCTCTAAGT	ATGAAGATGCTGGCGTGACA
CD206	TTCGGACACCCATCGGAATTT	CACAAGCGCTGCGTGGAT

## Data Availability

The data used to support the findings of this study are available from the corresponding author upon request.
